# *Astragalus polysaccharides* ameliorate epileptogenesis, cognitive impairment, and neuroinflammation in a pentylenetetrazole-induced kindling mouse model

**DOI:** 10.3389/fphar.2024.1336122

**Published:** 2024-02-09

**Authors:** Yuling Lu, Minglin Lin, Sijie Ou, Lanfeng Sun, Kai Qian, Huimin Kuang, Yuan Wu

**Affiliations:** ^1^ Department of Neurology, First Affiliated Hospital of Guangxi Medical University, Nanning, Guangxi Zhuang Autonomous Region, China; ^2^ Department of Colorectal and Anal Surgery, First Affiliated Hospital of Guangxi Medical University, Nanning, Guangxi Zhuang Autonomous Region, China

**Keywords:** *Astragalus polysaccharides*, epilepsy, cognitive deficit, network pharmacology, TLR4/NF-κB, neuroinflammation

## Abstract

**Background:** Epilepsy is a prevalent neurological disease where neuroinflammation plays a significant role in epileptogenesis. Recent studies have suggested that *Astragalus polysaccharides* (APS) have anti-inflammatory properties, which make them a potential candidate for neuroprotection against central nervous system disease. Nevertheless, the extent of their effectiveness in treating epilepsy remains enigmatic. Therefore, our study aims to investigate the potential of APS to mitigate epileptogenesis and its comorbidities by exploring its underlying mechanism.

**Methods:** Initially, we employed pentylenetetrazol-induced seizure mice to validate APS’ effectiveness. Subsequently, we employed network pharmacology analysis to probe the possible targets and signaling pathways of APS in treating epilepsy. Ultimately, we verified the key targets and signaling pathways experimentally, predicting their mechanisms of action.

**Results:** APS have been observed to disturb the acquisition process of kindling, leading to reduced seizure scores and a lower incidence of complete kindling. Moreover, APS has been found to improve cognitive impairments and prevent hippocampal neuronal damage during the pentylenetetrazole (PTZ)-kindling process. Subsequent network pharmacology analysis revealed that APS potentially exerted their anti-epileptic effects by targeting cytokine and toll-like receptor 4/nuclear factor kappa B (TLR4/NF-κB) signaling pathways. Finally, experimental findings showed that APS efficiently inhibited the activation of astrocytes and reduced the release of pro-inflammatory mediators, such as interleukin-1β (IL-1β), interleukin-6 (IL-6), and tumor necrosis factor-α (TNF-α). In addition, APS impeded the activation of the TLR4/NF-κB signaling cascade in a PTZ-induced kindling mouse model.

**Conclusion:** The outcomes of our study suggest that APS exerts an impact on epileptogenesis and mitigates cognitive impairment by impeding neuroinflammatory processes. The mechanism underlying these observations may be attributed to the modulation of the TLR4/NF-κB signaling pathway, resulting in a reduction of the release of inflammatory mediators. These findings partially agree with the predictions derived from network pharmacology analyses. As such, APS represents a potentially innovative and encouraging adjunct therapeutic option for epileptogenesis and cognitive deficit.

## 1 Introduction

Epilepsy is a prevalent chronic and debilitating neurological disease that manifests as spontaneous and recurrent seizures, affecting roughly 1% of the population. Recurring seizures are frequently accompanied by comorbidities, including cognitive impairment, psychiatric disorders, and behavioral abnormalities, resulting in a significant financial burden (Shen et al., 2022). To date, available treatments primarily aim to manage symptoms, yet up to 30% of epilepsy patients remain refractory to treatment and experience uncontrolled seizures (Brodie, 2016). Conventional anti-epileptic drugs are frequently linked to cognitive and behavioral adversities, highlighting the need for new drugs to prevent or treat epilepsy. Epileptogenesis is a chronic and complex phenomenon that involves the interaction of genetic and acquired factors, leading to an increased predisposition to seizures and ultimately resulting in the development of spontaneous recurrent seizures (Rong et al., 2019). It is noteworthy that the emergence of epileptogenesis is commonly accompanied by comorbid behavioral impairments, which are now recognized as essential endpoints for evaluating the efficacy of innovative therapeutic interventions (Terrone et al., 2016). Interventions targeting the anti-epileptogenic process may not only attenuate seizure onset, frequency, and severity but also ameliorate associated comorbidities and neuronal damage. These interventions, when implemented post-disease onset, may modify the course of the disease and confer disease-modifying effects (Dezsi et al., 2016). Hence, the pursuit of safe and efficacious anti-epileptogenesis drugs has emerged as an active area of medical research.

*Astragalus membranaceus* (AM), a botanical remedy with a long history of use in traditional Chinese medicine (TCM), has been recognized for its potential therapeutic effects. Previous studies have demonstrated that AM extract has the potential to provide anti-convulsant effects by protecting against oxidative damage and mitochondrial dysfunction (Aldarmaa et al., 2010). AM contains components such as *Astragalus mongholicus* Bunge saponin extract and baicalin, which activate the TLR4/MYD88/Caspase-3 pathway and exert anti-convulsant and neuroprotective effects in PTZ-induced epileptic animal models (Jalsrai et al., 2010; Yang et al., 2021). In addition, *Astragalus polysaccharides* (APS), key active components of AM, have multiple pharmacological effects, including anti-inflammatory, immune regulation, and antitumor properties (Zheng et al., 2020). Polysaccharides have also been identified as a potential agent for treating epilepsy (Xie et al., 2022). Therefore, APS may also play a crucial role in epileptogenesis and comorbidities. Nevertheless, the therapeutic potential and underlying mechanism of APS in epileptogenesis and comorbidities remain unexplored.

The management of epileptogenesis and its comorbidities remains challenging, highlighting the need for innovative and effective adjunct therapies. A promising candidate is APS, a natural herbal extract with potential anti-inflammatory and neuroprotective properties. In this study, we assessed the potential effects of APS through continuous administration in pentylenetetrazole (PTZ)-induced epileptic models and investigated the action of APS during the progression of PTZ-induced kindling and the cognitive function of the mice. To further elucidate the mechanism of APS in epileptogenesis and associated cognitive impairment, network pharmacology analysis was performed, and the identified key targets and signaling pathways were subsequently validated in the PTZ-induced kindling mouse model. This study highlights the potential of APS as a novel and encouraging adjunct therapeutic option for epileptogenesis and its associated comorbidities.

## 2 Materials and methods

### 2.1 Animals

Experimental Animal Center of Guangxi Medical University provided male SPF C57BL/6 mice (5–8 weeks old, 20–25 g) with license number SCXK(Guangxi)2014-0003. Mice were kept for 7 days under standard temperature, humidity, and lighting conditions before the animal experiment. We provided the mice with *ad libitum* access to food and water throughout the study. NIH’s Guide for the Care and Use of Laboratory Animals was followed strictly during the experimental procedures. Approval for all animal experiments was obtained from the Ethics Committee of the First Affiliated Hospital Guangxi Medical University (ethical approval number: 2023-E162-01). All measures were taken to ensure minimal discomfort, minimal suffering, and the use of the minimum number of animals possible.

### 2.2 Drug administration protocol

APS was procured from Solarbio, Beijing, China (Catalog No. A7970), exhibiting a polysaccharide content exceeding 90% (by mass) (Zhao et al., 2023). PTZ was sourced from Sigma-Aldrich, United States. APS was solubilized in deionized water, while pentylenetetrazol was dissolved in 0.9% saline. The control group mice received oral administration of deionized water in an equivalent volume to APS. For the acute study, APS was administered at doses of 200, 300, 400, 500, 600, and 800 mg/kg orally per d for a duration of 7 days. In the PTZ-kindling model, a daily dosage of 400 mg/kg APS was administered until the development of kindling. All substances were administered in a 10 mL/kg volume.

### 2.3 Experimental design

**FIGURE 1:**
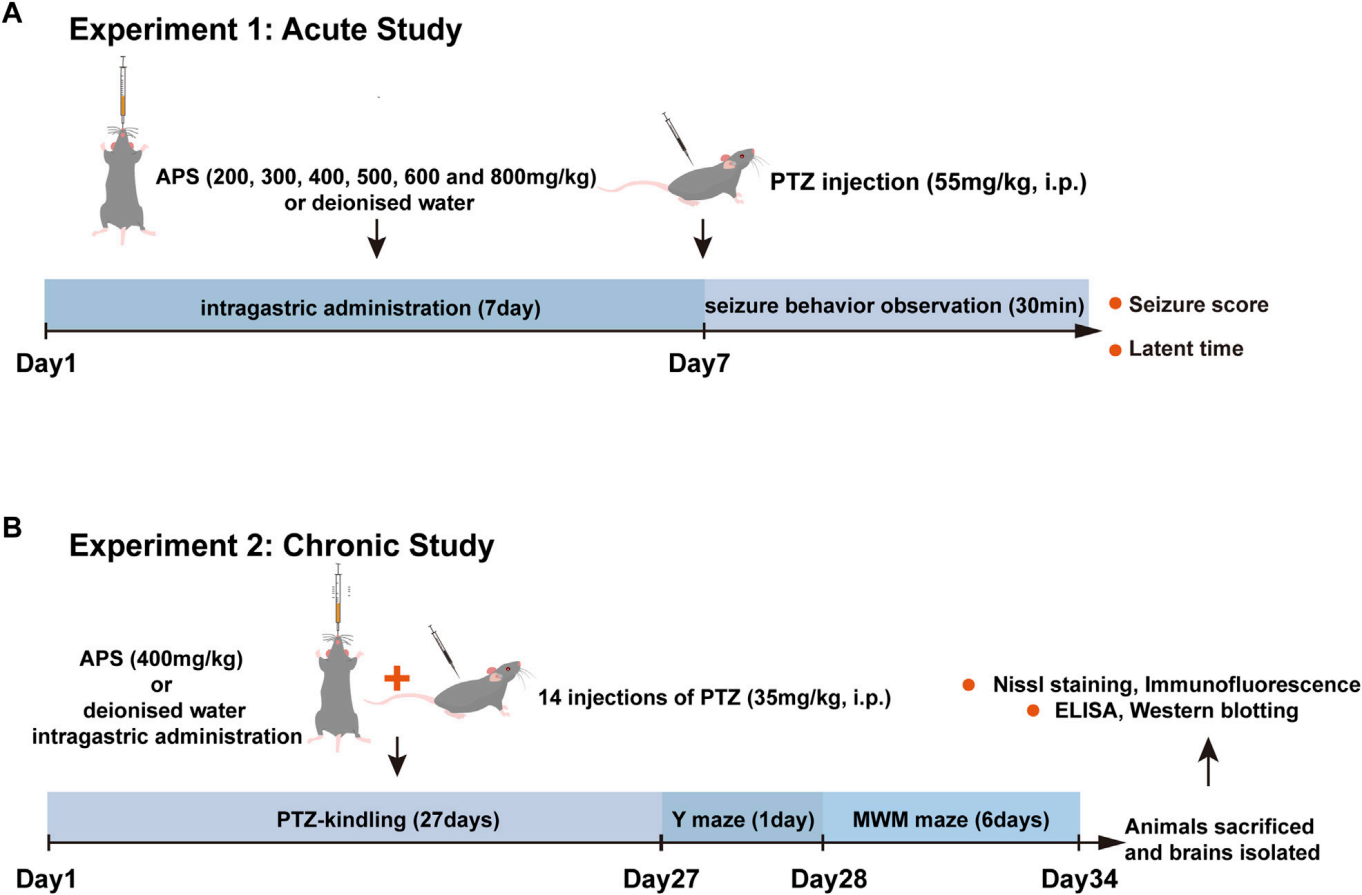
The flow chart of experimental design. **(A)** Assessment of the impact of APS (200, 300, 400, 500, 600, and 800 mg/kg) on PTZ-induced acute seizures. The subjects were orally administered deionized water or APS daily for seven consecutive days, and on the 7th day, 1 h after drug administration, PTZ (55 mg/kg) was intraperitoneally injected. Subsequently, the latency period and seizure score were measured and recorded for 30 min post-injection. **(B)** Evaluation of the effects of APS (400 mg/kg) on PTZ-induced chronic seizures. For kindling induction, animals received 14 PTZ injections on alternate days. Treatment groups received daily APS administration (400 mg/kg) from the 1st day of PTZ administration until the last day (27th day). Following the kindling process, mice underwent behavioral assessment for cognition, followed by brain isolation for biochemical and histopathological evaluation. Abbreviations: PTZ, Pentylenetetrazole; MWM, Morris water maze; ELISA, Enzyme-linked immunosorbent assay.

Our experimental design comprises two components: the first part investigates the impact of APS on the acute seizure model, while the second examines its effects on the chronic kindling model induced by PTZ. The detailed experimental design is shown in Figure 1.

#### 2.3.1 Experiment 1: acute study

##### 2.3.1.1 Acute seizure model induced by PTZ

Mice were systematically divided into eight distinct groups, namely, the control group, PTZ group, and six APS groups, to evaluate the impact of APS on the acute seizure model induced by PTZ (Hoda et al., 2021). They were administered oral doses of deionized water and 200, 300, 400, 500, 600, or 800 mg/kg of APS to each group for seven consecutive days, respectively. On the 7th d of the dosing protocol, following a 1 h interval post-drug administration, PTZ (55 mg/kg) was injected into the mice intraperitoneally, and their resultant latency period and seizure scores were measured and recorded for 30 min post-injection.

##### 2.3.1.2 Seizure observation

In both acute seizure model and chronic kindling model induced by PTZ, seizures were scored using a modified Racine’s scale (Luttjohann et al., 2009) that comprised the following stages: Stage 0—the absence of any behavioral response; Stage 1—the subject displays immobility; Stage 2—the organism displays small tics throughout its entire body, with the potential for the animal to exhibit a state of immobility or not; Stage 3—clonus in either one or both of the forelimbs; Stage 4—clonic seizures in both forelimbs; Stage 5—generalized tonic-clonic seizures characterized by postural collapse, accompanied by wild jumping or running; Stage 6—extensive hind limb straining leading to death.

#### 2.3.2 Experiment 2: chronic study

##### 2.3.2.1 PTZ-induced chronic kindling model

In the latter phase of the study, the effects of APS on a chronic epilepsy model in mice induced by the PTZ-kindling method were evaluated. The preliminary experiment established that APS at a 400 mg/kg dose exhibited significant suppression of PTZ-induced acute seizures. Consequently, the efficacy of the 400 mg/kg dose was examined selectively in the subsequent subchronic test. The mice were allocated to the following groups:

1) Control group: Mice were administered deionized water orally for 27 consecutive days and were alternatively intraperitoneally infused with 0.9% saline.
2) PTZ group: Mice were continually administered deionized water orally for 27 consecutive days and were alternatively intraperitoneally injected with PTZ (35 mg/kg).
3) APS group: Mice were given 400 mg/kg of APS orally for 27 consecutive days and were injected with PTZ (35 mg/kg) intraperitoneally once every other days.

The seizure scores were monitored for 30 min post-PTZ injection. When animals attained a seizure score of 4- or higher in three consecutive PTZ administrations, they were deemed fully kindled (Li et al., 2022). The full kindling rate is computed by dividing the number of mice achieving full kindling by the total number of mice.

##### 2.3.2.2 Y-maze test

Y-maze evaluation was performed following established protocols (Shen et al., 2020). On the 28th d of the experiment, the Y-maze test was performed using a platform of three arms, each measuring 30 cm long, 6 cm wide, and 15 cm, which were arranged to match the natural inclination of mice towards exploring new environments. The three arms were labeled as the start arm, novel arm, and other arm in a randomized manner. Before the test trial, mice were allowed to explore only two arms during the training phase, while the third arm remained inaccessible. During the test trial, which lasted for 5 min, all three arms were accessible, and mice were given 1 h to rest. The Smart 3.0 system (Panlab Harvard Apparatus, Spain) automatically recorded all test sessions.

##### 2.3.2.3 Morris water maze (MWM) assessment

Declarative learning and memory in mice were investigated through the MWM test, adhering to a previously established protocol (Wang et al., 2021) and conducted subsequent to the Y-maze experiment. The experiment was conducted in a water tank measuring 120 cm in diameter and 50 cm in height, filled with water maintained at a temperature of 23°C ± 1°C. A transparent circular platform, 10 cm in diameter, was placed within the pool to facilitate the mouse’s escape from the water. The pool, divided into four quadrants, featured a circular platform positioned in the first quadrant (target quadrant). Strategically positioned distal cues encircled the pool and remained consistently in place across all testing sessions. The MWM assessment spanned 6 consecutive days, encompassing hidden platform trials over a 5-day period, succeeded by a single d designated for probe trials. During the hidden platform trials, each mouse underwent training once daily from each of the four quadrants, with a 30-min interval between adjacent trials, for a consecutive period of 5 days. During the spatial probe test, mice were positioned in the third quadrant and allowed to swim for 60 s to assess spatial memory. The swimming trajectory data were acquired using video-based motion tracking software (SMART 3.0, Panlab Harvard Apparatus, Spain).

##### 2.3.2.4 Tissue harvest

After conducting MWM tests, the animals were anesthetized with isoflurane. Five randomly selected individuals from each group had their right brains fixed in 4% paraformaldehyde, embedded in paraffin and sectioned for Nissl staining and immunofluorescence analysis. The remaining brain tissues were promptly dissected to isolate the cortex and hippocampus, which were rapidly preserved in liquid nitrogen. The cortex and hippocampus were stored at −80°C until further analysis.

### 2.4 Network pharmacological analysis

First, the GenenCards database was utilized to predict APS targets, and the keywords “epilepsy” and “cognitive impairment” were retrieved from the same database to extract relevant pathogenic targets. The overlapping genes were then identified and visualized in a Venn diagram using the Draw Venn Diagram website (https://bioinformatics.psb.ugent.be/webtools/Venn/). Subsequently, protein-protein interaction (PPI) analysis was conducted on those interaction genes that met the threshold of a confidence score of >0.9 and species as “*Homo sapiens*” in the STRING database (https://cn.string-db.org/). Based on MCC algorithms, the top 10 core targets were selected using Cytoscape 3.8.2 software with a cytoHubba plugin. Finally, the overlapping targets were subjected to gene ontology (GO) and Kyoto Encyclopedia of Genes and Genomes (KEGG) enrichment analysis using the DAVID database (https://david.ncifcrf.gov/), and the top 10 GO terms and top 20 signaling pathways were visualized in bar plots with the aid of bioinformatics tools.

### 2.5 Nissl staining

Following the preparation of 4-μm coronal sections of mouse hippocampal tissues, the sections were initially hydrated with gradient alcohol, washed thrice using distilled water, and incubated with toluidine blue (Servicebio, G1032) for 2–5 min. The Nissl staining protocol in this study followed previously established procedures (Xiang et al., 2021). The sections were then subjected to differentiation using 95% ethanol until a clear background was achieved. The slides were cleared employing xylene and were subsequently sealed with neutral gum. The neuronal damage was subsequently analyzed by a microscope (Olympus Corporation, Tokyo, Japan). A blinded observer quantitatively analyzed the hippocampus region’s dark neurons by counting labeled cells in three randomly selected regions from sections from each mouse (*n* = 5/group). Dark neurons were quantified using the multi-point tool in ImageJ software (NIH, Bethesda, MD, United States).

### 2.6 Immunofluorescence

The 4-μm coronal sections of mouse hippocampal tissues were dewaxed in xylene and dehydrated in graded ethanol solutions. Antigen retrieval was achieved by heating the slides in a sodium citrate buffer solution for 20 min. Subsequent steps included a 20-min incubation in 3% hydrogen peroxide (South Land Pharmaceutical, Guangdong, China, 180303) and an hour-long incubation in 5% bovine serum albumin (Beyotime, Shanghai, China, ST023). For dual immunofluorescence staining of GFAP/C3, tissue sections were incubated overnight at 4°C with anti-GFAP (mouse monoclonal, 1:200, Servicebio, GB12096) and anti-C3 (rabbit monoclonal, 1:500, Abcam, ab200999). This was followed by a 1-h incubation at 4°C with Alexa Fluor 488-conjugated goat anti-mouse IgG (1:400, Servicebio, GB25301) and Cy3-conjugated goat anti-rabbit IgG (1:300, Servicebio, GB21303). A similar procedure was applied for IBA1/CD68 staining, using anti-IBA1 (rabbit polyclonal, 1:500, Servicebio, GB113502) and anti-CD68 (rabbit polyclonal, 1:3000, Servicebio, GB113109), followed by incubation with Alexa Fluor 488-conjugated goat anti-rabbit IgG (1:400, Servicebio, GB25303) and Cy3-conjugated goat anti-rabbit IgG (1:300, Servicebio, GB21303). Following triple washes in PBS, the cell nuclei were counterstained using DAPI. The processed samples were then analyzed and captured with a panoramic Midi scanner from 3DHISTECH (Budapest, Hungary). Quantitative cell counts in the hippocampal region were performed on three randomly selected fields from sections per mouse (*n* = 5/group), conducted by an observer blind to the experimental conditions. CD68^+^/IBA1^+^positive cells were identified by overlapping red CD68 and green IBA1 labeling, while C3^+^/GFAP^+^ positive cells were marked by overlapping red C3 and green GFAP labeling. The counting of positive cells was facilitated using the multi-point tool in ImageJ software (Meyer et al., 2023).

### 2.7 Enzyme-linked immunosorbent assay (ELISA)

Upon dissection of the mouse brains, the cortex and hippocampal tissues were promptly extracted under chilled conditions. To obtain the supernatant, the tissues mentioned above were homogenized in PBS and subjected to centrifugation at 5,000 rpm for 15 min at 4°C. Commercially available ELISA kits (Solarbio, Beijing, China) were employed to measure the levels of interleukin-1β (IL-1β), tumor necrosis factor-α (TNF-α), and interleukin-6 (IL-6) (Catalog No: SEKM-0002, SEKM-0034 and SEKM-0007, respectively) in the cortex and hippocampal tissues of mice, as per the manufacturer’s instructions (Kan et al., 2021).

### 2.8 Western blotting

To extract total proteins, hippocampal tissues were homogenized with radioimmunoprecipitation assay buffer (R0010, Solarbio, Beijing, China) (Luo Y. et al., 2023) containing 1% protease and phosphatase inhibitors (P1260, Solarbio). The concentration of protein was determined utilizing the bicinchoninic acid protein assay kit (AR0146, Boster, Wuhan, China) (Luo XD. et al., 2023), followed by separation of the protein via 10% sodium dodecyl sulfate-polyacrylamide gel electrophoresis gels (PG112, Epizyme Biotech, Shanghai, China) and transferring it onto nitrocellulose (NC) membranes (0.45 μm; Merck, Germany) for 25–30 min in rapid transfer buffer (WB4600, NCM Biotech, Suzhou, China). After blocking with 5% bovine serum albumin (SW3015, Solarbio), the NC membranes underwent incubation with primary antibodies, including TLR4 (1:1000, Servicebio, GB11519), NF-κB p65 (1:1000, Cell Signaling Technology, 8242), Phospho-IκBα (Ser32) (1:1000, Cell Signaling Technology, 2859), and β-Tubulin (1:1000, Servicebio, GB11017), overnight at 4°C. Following this step, the NC membranes were incubated at room temperature for 1 h with IRDye 800CW goat anti-rabbit IgG (H+L) (1:10000, LICOR, D11215-03). Subsequently, protein bands were visualized using an Odyssey Infrared Imaging System (LI-COR Biosciences), and band density was quantified using ImageJ software (NIH, Bethesda, MD, United States), with β-tubulin as an internal reference.

### 2.9 Statistical analysis

**FIGURE 2:**
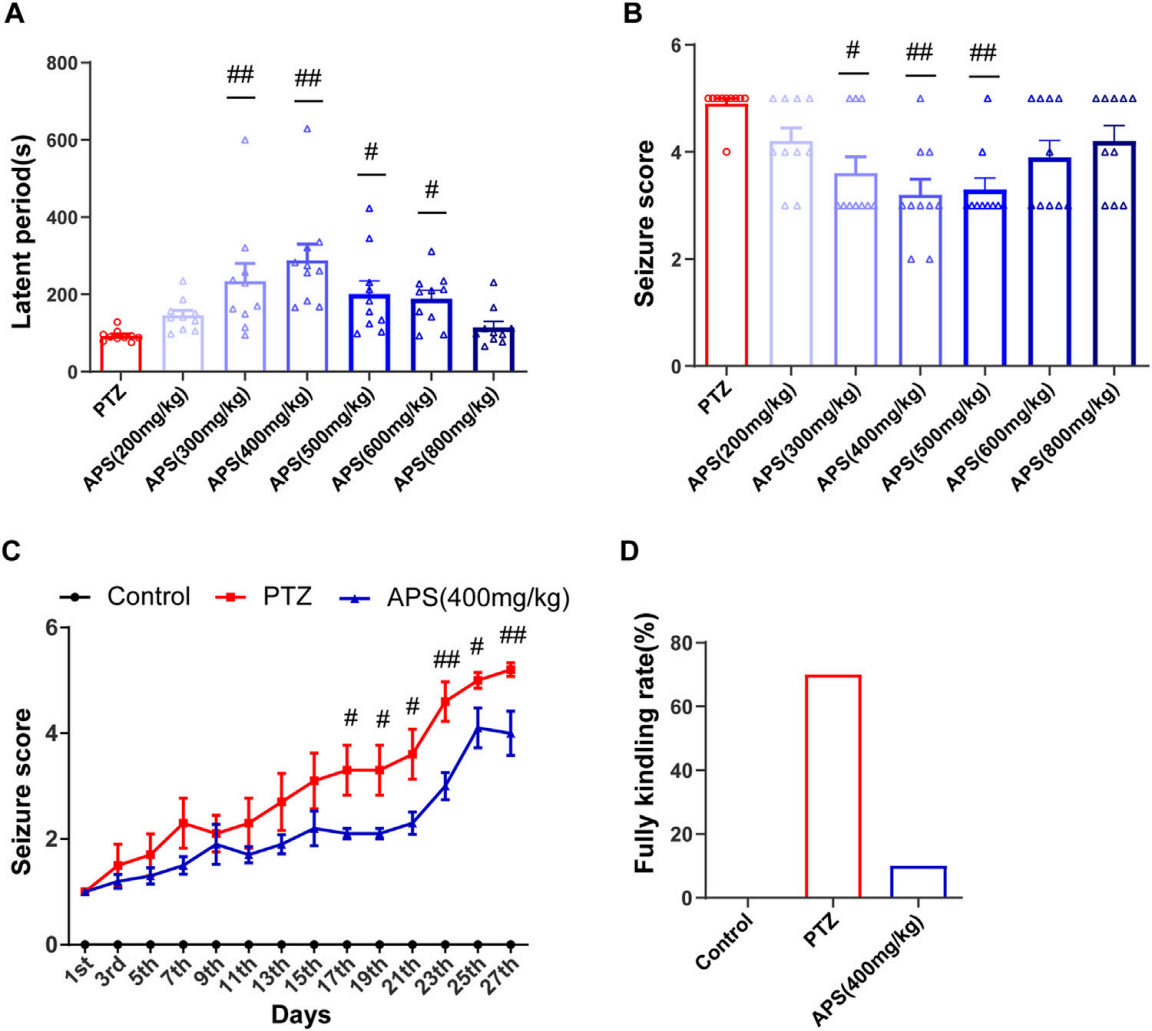
Effects of APS on PTZ-induced acute seizures and PTZ-kindling process. **(A)** The latent period of PTZ-induced acute seizures in mice treated with deionized water or APS (200, 300, 400, 500, 600, and 800 mg/kg). **(B)** Seizure score of PTZ-induced acute seizures in mice treated with deionized water or APS (200, 300, 400, 500, 600, and 800 mg/kg). **(C)** Seizure score during the PTZ-kindling process. **(D)** APS (400 mg/kg) decreased the rate of complete kindling. **(A,B)** were analyzed using nonparametric tests, including the Kruskal-Wallis one-way ANOVA test. **(C)** was analyzed using a repeated two-way ANOVA model followed by Bonferroni *post hoc* test. Data are shown as mean ± SEM (*n* = 10 mice/group). ^#^: *p* < 0.05, ^##^: *p* < 0.01 for PTZ group *vs*. APS group.

We utilized GraphPad Prism software (version 8.0) for graphical data representation. Data analysis was conducted using SPSS software (version 25.0). The measurement data were reported as the mean ± standard error of the mean (x ± SEM). To determine whether or not the data were normally distributed, we employed the Shapiro-Wilk test. For normally distributed datasets, the one-way analysis of variance (ANOVA) followed by Tukey’s *post hoc* analysis (for equal homogeneity) or Dunnett’s T3 test (for unequal homogeneity) was conducted to assess the differences between groups. Nonparametric tests were conducted to estimate the non-normally distributed data. A repeated two-way ANOVA model, followed by Bonferroni *post hoc* tests, was conducted to analyze the kindling, and MWM test data were presented in Figures 2C, 4A, B. Statistical significance was considered *p* < 0.05.

## 3 Results

### 3.1 APS exerted significant inhibition of acute seizures induced by PTZ

To assess the efficacy of APS and determine the optimal concentration in an acute epilepsy model, we pre-administered varying concentrations of APS as an intervention in a PTZ-induced acute seizure model in mice (Figure 1A). These groups included the control group, PTZ group, and six APS-treated groups with doses of 200, 300, 400, 500, 600, and 800 mg/kg. The control group did not exhibit epileptic seizures. Subsequent to APS intervention, a significant prolongation of seizure latency periods was observed, forming a bell-shaped curve across the APS dose range (200, 300, 400, 500, 600, and 800 mg/kg). Notably, the APS (400 mg/kg) group exhibited the most pronounced extension in seizure latency, with statistically significant differences observed (*p* < 0.01, Figure 2A). In contrast, the APS (200 and 800 mg/kg) groups showed a modest increase in seizure latency, but these changes did not reach statistical significance. Following APS intervention, a substantial reduction in seizure scores was noted, depicting a U-shaped dose-response curve covering the APS dosage range (200, 300, 400, 500, 600, and 800 mg/kg) (Figure 2B). Specifically, the APS (400 mg/kg) group displayed the most significant reduction in seizure scores, reaching statistical significance (*p* < 0.01). Conversely, seizure scores were reduced in the APS (200, 600, and 800 mg/kg) groups, although these changes did not achieve statistical significance. These findings collectively suggest a dose-dependent and nonlinear effect of APS on seizure latency periods and seizure scores, with the most significant impact observed at the 400 mg/kg dosage. This outcome underscores the potential anti-epileptic properties of APS in the PTZ-induced acute seizure model.

### 3.2 APS lessened seizure severity during the PTZ-kindling process in mice

The administration of APS at a dose of 400 mg/kg demonstrated notable anti-convulsant effects in PTZ-induced acute seizure models. Upon observing the promising results, we aimed to determine the potential impact of APS (400 mg/kg) on epileptogenesis by conducting a long-term PTZ-kindling process on mice throughout 27 days (Figure 1B). The progression of PTZ-induced kindling was monitored by recording the modified Racine score in mice every alternate d over a 27-day period, which encompassed 14 injections. Upon analysis, it was detected that the administration of 400 mg/kg APS had no significant impact on the body weight of mice during the PTZ-kindling process compared with the control and PTZ groups (Supplementary Figure S1). The APS group administered with a dose of 400 mg/kg demonstrated a decrease in seizure scores on d 17, 19, 21, 23, 25, and 27, as depicted in Figure 2C. Moreover, APS treatment at the same dosage resulted in a lower fully kindling rate (10%) after 14 injections of PTZ compared to the PTZ alone group (70%), as illustrated in Figure 2D. These data underscore the possible anti-epileptogenic characteristics of APS that have been noted during the mice’s PTZ-kindling procedure.

### 3.3 APS improved recognition in the Y-maze and MWM tasks

**FIGURE 3:**
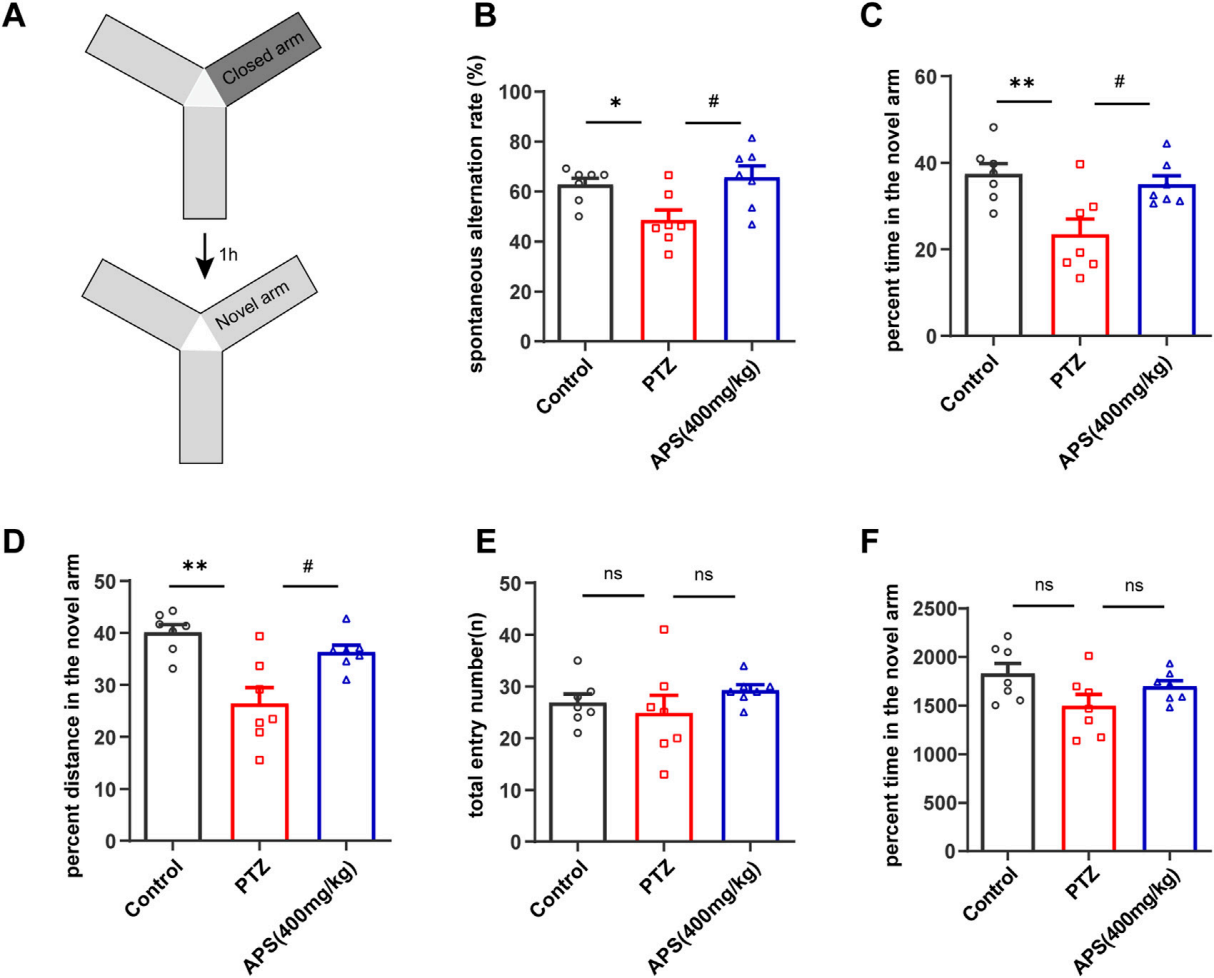
The behavioral response of the mice was evaluated by the Y-maze test. **(A)** A depiction of the Y-maze and a schedule outlining the progression of the training and testing phases. **(B)** Spontaneous alternation rate. **(C,D)** The percentage of time and distance in the novel arm throughout the testing period. **(E,F)** Total entry number and total distance during the testing phases. Figures 3B, C, E, F were analyzed using one-way ANOVA with Tukey’s *post hoc* multiple comparison tests. Figure 3B was subjected to one-way ANOVA with Dunnett’s T3 *post hoc* multiple comparison test. Data were presented as mean ± SEM (*n* = 7 mice/group). *: *p* < 0.05, **: *p* < 0.01 for PTZ group *vs*. control group; ^#^: *p* < 0.05 for PTZ group *vs*. APS group.

To assess the impact of the test drugs on the short-term recognition capacity of the animal models, we employed the Y-maze (Figure 3A). The results obtained from the Y-maze test revealed a substantial reduction in the spontaneous alternation rate of the PTZ group of mice as compared to the control group (Figure 3B, *p* < 0.05). In addition, the PTZ group displayed a lower preference toward exploring the novel arm, spending an average of 23.4% ± 3.6% of their total exploration time when compared to the control group, which spent 37.4% ± 2.4% (Figure 3C, *p* < 0.01). Moreover, the percentage of distance traveled by the PTZ group in the novel arm was observed to be less (26.4% ± 3.1%) in comparison with the control group (40.1% ± 1.5%) (Figure 3D, *p* < 0.01). These findings provide evidence of impaired spatial working memory in the PTZ group compared to the control group. It is noteworthy that the mice of the APS group depicted a significant rise in the spontaneous alternation rate (65.7% ± 4.6%) after the APS treatment administration, which surpassed that of the PTZ group (48.5% ± 4.0%, *p* < 0.05). Furthermore, the APS group exhibited a higher average percentage of time spent exploring the novel arm (35.0% ± 2.0%) in contrast to the PTZ group (23.4% ± 3.5%, *p* < 0.05). Similarly, the APS group also displayed a higher mean percentage of distance covered in the novel arm (36.3% ± 1.3%), which was 1.4 times more than that observed in the PTZ group (26.4% ± 3.1%, *p* < 0.05). The statistical analysis indicated no noticeable differences in the total entry number and total distance between the groups, suggesting that the mice mentioned above exhibited no motor impairments (Figures 3E, F).

**FIGURE 4:**
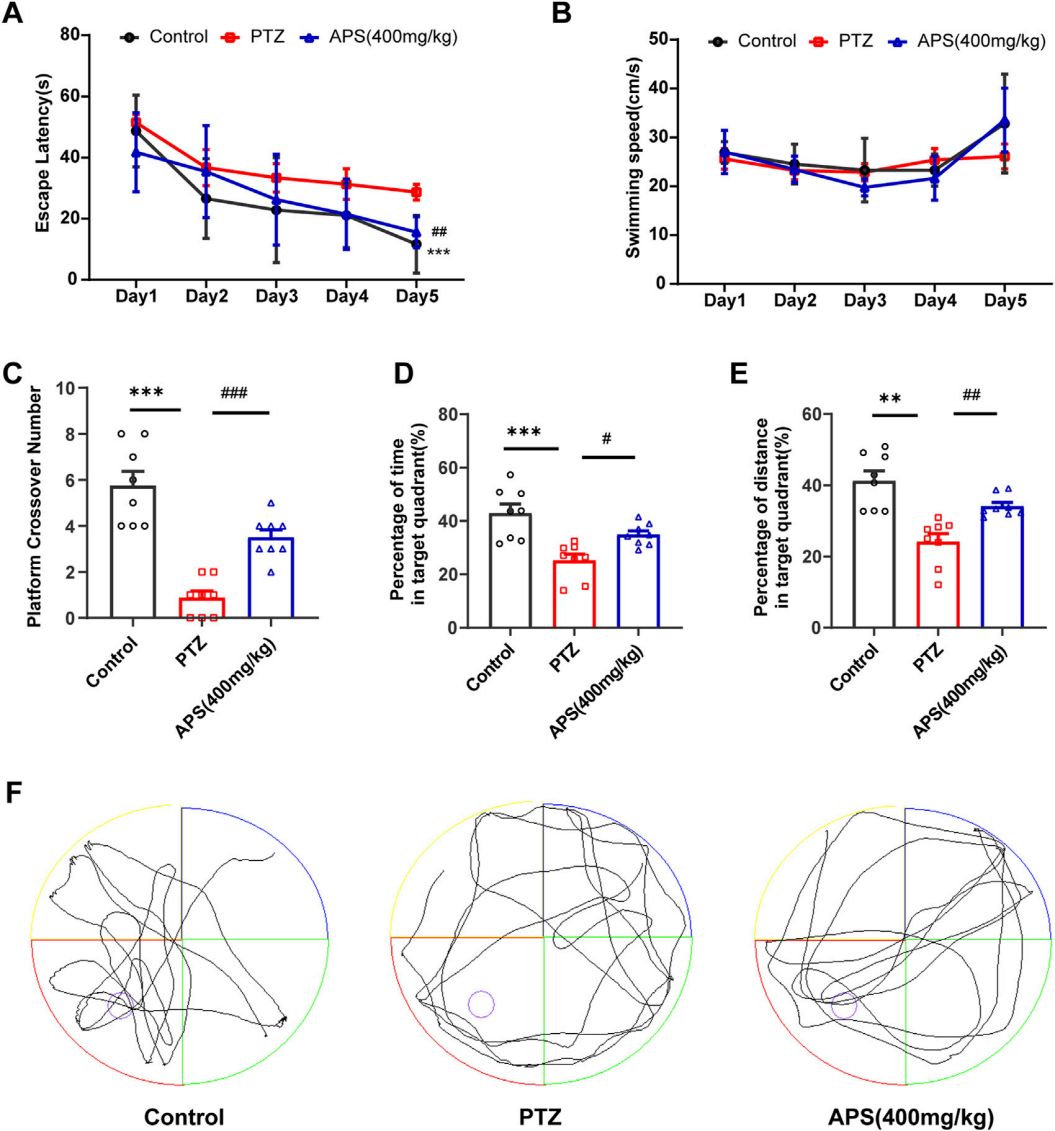
The performance of mice in the Morris water maze (MWM) test. **(A)** The escape latencies of mice in different groups during five consecutive training days. **(B)** The swimming speeds of mice in different groups during five consecutive training days. **(C)** The average crossing number over the platform during the probe test. **(D)** The percentage of time spent in the target quadrant during the probe test. **(E)** The percentage of distance in the target quadrant during the probe test. **(F)** Representative swimming paths of mice in the three groups during the probe trial. Figures 4A, B was analyzed using a repeated two-way ANOVA model followed by the Bonferroni *post hoc* test. Figures 4C, F was subjected to one-way ANOVA with Dunnett’s T3 *post hoc* multiple comparison test. Figure 4D was subjected to one-way ANOVA with Tukey’s *post hoc* multiple comparison test. Data were presented as mean ± SEM (*n* = 8 mice/group). **: *p* < 0.01, ***: *p* < 0.001 for PTZ group *vs*. control group; ^#^: *p* < 0.05, ^##^: *p* < 0.01, ^###^: *p* < 0.001 for PTZ group *vs*. APS group.

Subsequently, we conducted an MWM test to evaluate the impact of APS on spatial learning and memory of PTZ kindling mice. As shown in Figure 4A, the escape latency for all groups exhibited a gradual decrease throughout the 5-day hidden platform trial. On the 5th day of training, the mice in the PTZ group demonstrated a significantly prolonged escape latency compared to the control group (*p* < 0.001). Administration of APS at a dosage of 400 mg/kg resulted in a significant reduction in escape latency to locate the hidden platform. Notably, no significant differences in swimming speed were observed among all experimental groups (Figure 4B). In the probe test, where the platform was absent, the mice in the PTZ group exhibited fewer crossings of the original platform position and spent less time in the target quadrant compared to control mice, indicating a decline in memory within the PTZ-induced kindling mouse model. Concurrently, APS treatment significantly mitigated memory impairment in PTZ-induced kindling mice, as evidenced by an increased number of crossings at the original platform position, elevated % distance, and % time spent in the target quadrant (Figures 4C–E). Moreover, Figure 4F illustrates the characteristic swimming paths of different groups in the probe test on the 6th day. Collectively, these findings underscore the notable capacity of APS treatment to ameliorate cognitive deficits in PTZ-induced kindling mice.

### 3.4 APS attenuated the damage of neurons induced by PTZ kindling in the hippocampus (Hip)

**FIGURE 5:**
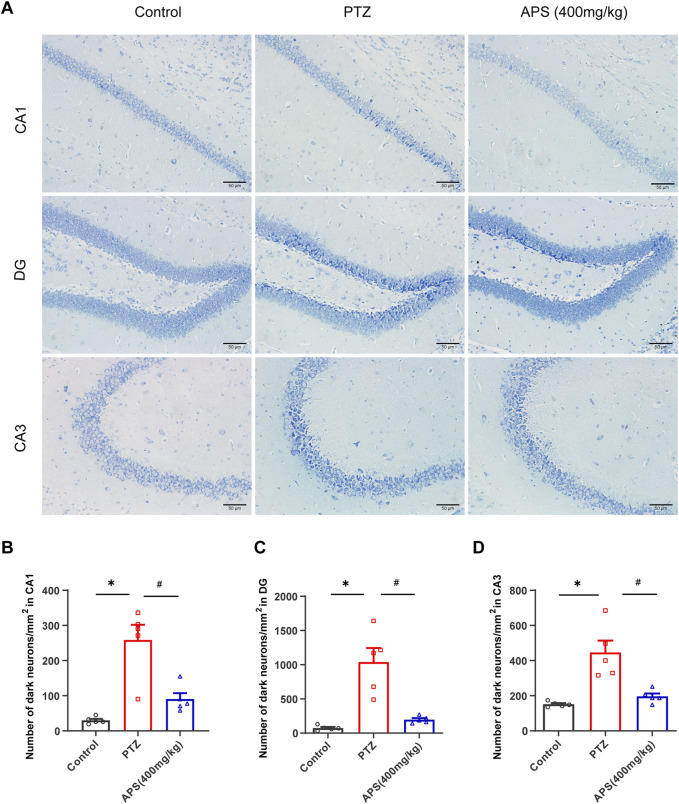
APS attenuates the damage of neurons in the Hip during the progression of PTZ-induced kindling. **(A)** Representative photomicrographs were obtained using Nissl staining to show that APS prevented PTZ-induced damage to hippocampal neurons in the CA1, DG, and CA3 areas following kindling. **(B–D)** Quantitative analysis of the number of dark neurons/mm^2^ in CA1, DG, and CA3 regions. Figure 5B was subjected to one-way ANOVA with Tukey’s *post hoc* multiple comparison test. Figures 5C, D was subjected to one-way ANOVA with Dunnett’s T3 *post hoc* multiple comparison test. Data are shown as mean ± SEM (*n* = 5 mice/group). *: *p* < 0.05 for PTZ group *vs*. control group; ^#^: *p* < 0.05 for PTZ group *vs*. APS group. Scale bars = 50 μm.

Our investigation also included an analysis of the impact of APS on the neuronal damage of the Hip induced by PTZ-induced kindling. Histological analysis was conducted using Nissl staining to evaluate the hippocampal regions. As illustrated in Figure 5, the CA1 (Cornu Ammonis 1), DG (Dentate Gyrus), and CA3 (Cornu Ammonis 3) regions of the Hip of the PTZ group showed a considerable increase in Nissl-stained dark neurons relative to those observed in the control group. The utilization of APS in PTZ-induced kindling reduced the number of dark neurons in the Hip. As depicted in Figure 5, APS treatment resulted in a substantial reduction in the number of dark neurons noted in the hippocampal CA1, DG and CA3 regions when contrasted with the PTZ group (CA1: 90.5 ± 17.0 *vs*. 258.5 ± 43.3, *p* < 0.05; DG: 196.5 ± 22.8 *vs*. 1039.2 ± 205.1, *p* < 0.05; CA3: 196.5 ± 16.8 *vs*. 445.9 ± 67.9, *p* < 0.05).

### 3.5 Network pharmacological construction and analysis

**FIGURE 6:**
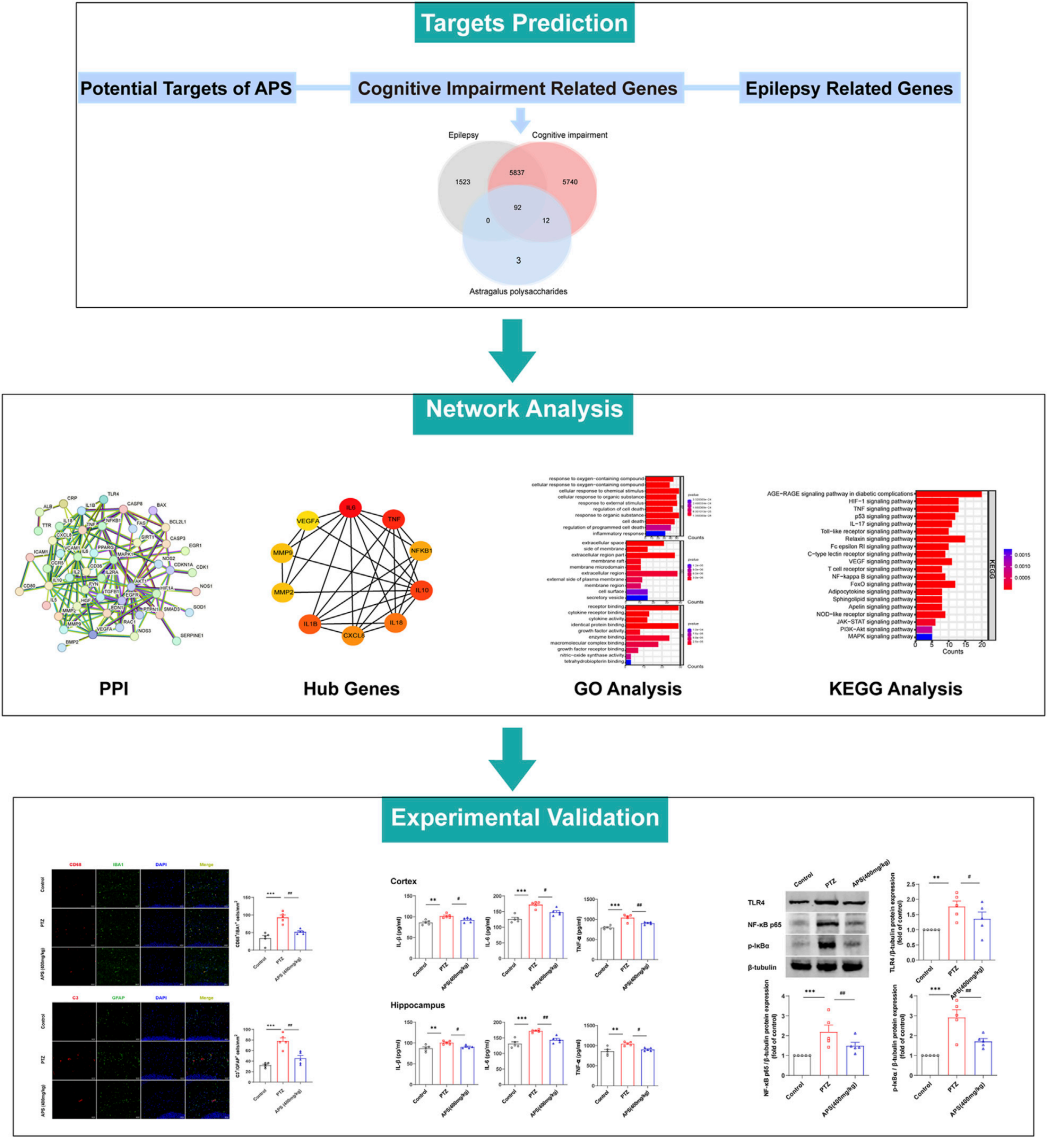
The flow chart of this study based on network pharmacology.

**FIGURE 7:**
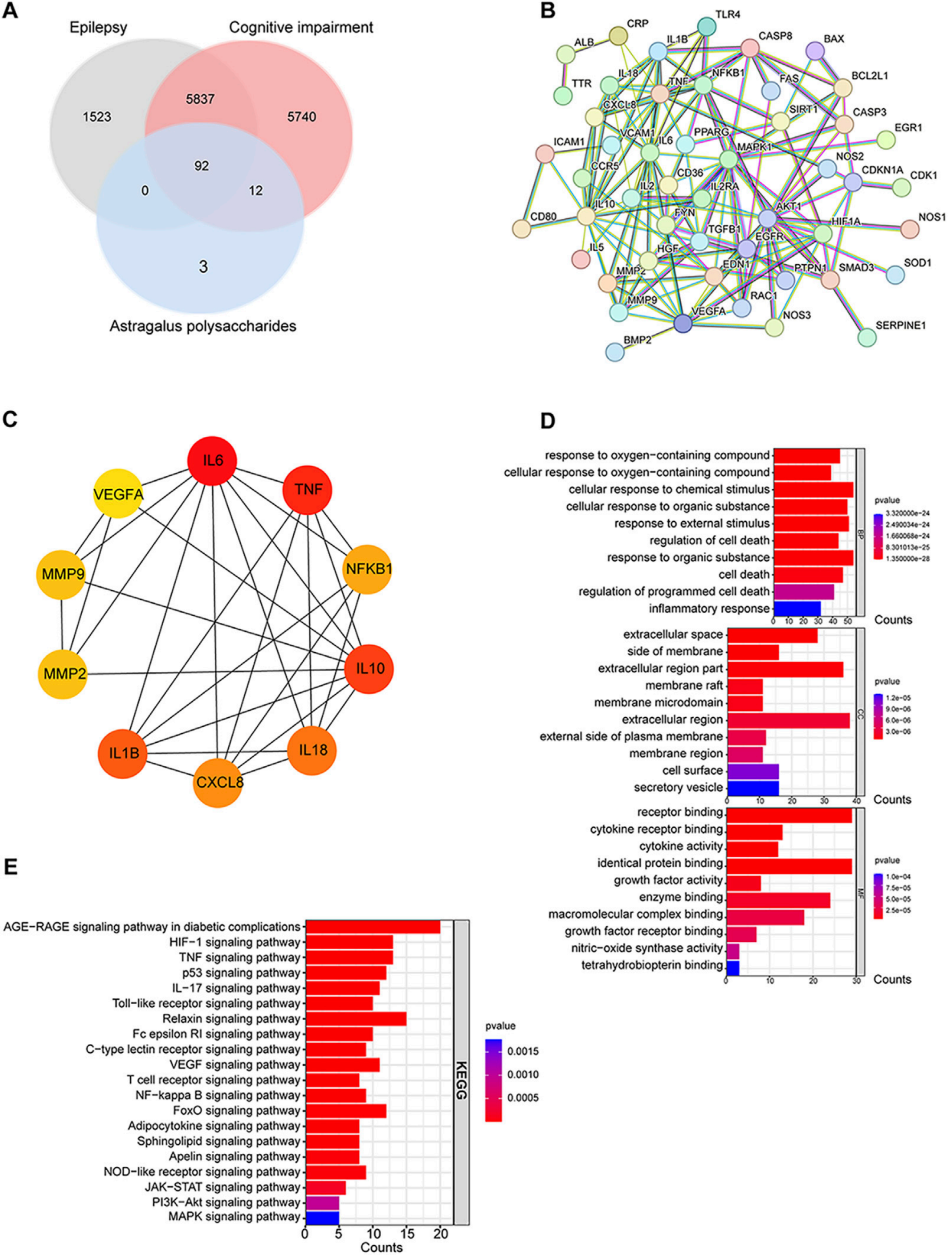
Network construction and analysis for APS, epilepsy, and cognitive impairment. **(A)** Venn diagram of APS, epilepsy, and cognitive impairment. **(B)** PPI network of common targets. **(C)** Top 10 hub gene analysis. **(D)** GO functional enrichment analysis of intersecting targets. From top to bottom: top 10 biological process (BP) subtype histogram, cellular component (CC) subtype histogram, and molecular function (MF) subtype histogram. **(E)** Results of KEGG pathway enrichment analysis.

To delve into the potential mechanisms and key targets underlying APS’s efficacy in epilepsy treatment, we performed a network pharmacology analysis on the action targets of its drug components and the pathogenic targets of the disease (Figure 6). Based on the GeneCards database, we obtained 107 targets of APS, 7,452 targets of epilepsy, and 11,681 targets of cognitive impairment. Finally, 92 targets overlapped (Table 1, Figure 7A). The PPI network was visualized by the online STRING database using the overlapping targets (Figure 7B). There were 49 protein nodes and 141 edges in the network. Herein, the top 10 core genes, including IL6, TNF, IL1B, IL10, IL18, CXCL8, NF-κB1, MMP2, MMP9, and VEGFA, were identified using the plugin “cytoHubba” according to MCC algorithms (Figure 7C). The nodes in the network represented the potential targets, and the color depth was proportional to the MCC values. The connecting lines between the nodes indicated the potential interaction relationship between the targets. GO enrichment analysis of the intersection targets was performed using the DAVID database according to *p* < 0.05 and FDR < 0.05. A total of 1,831 items of biological processes (BP), 18 items of cellular components (CC), and 17 items of molecular function (MF) were obtained. The top 10 terms of BP, CC, and MF are listed in Figure 7D. The outcomes of the KEGG pathway enrichment analysis revealed 109 enrichment pathways. Irrelevant pathways were removed, and then the top 20 signaling pathways were selected based on their *p*-values as the main signaling pathways, such as the Toll-like receptor signaling pathway, HIF-1 signaling pathway, TNF signaling pathway, and AGE-RAGE signaling pathway (Figure 7E).

**TABLE 1:** The overlapping 92 targets.

IL18	EDN1	CDK1	HGF	EGFR	CERNA3	TRP-AGG2-5
FAS	ALB	EGR1	NOS1	CDKN1A	SOD2-OT1	RAB4B-EGLN2
TLR4	CRP	MIR204	BGLAP	CASP3	LINC02605	TMX2-CTNND1
TTR	ODC1	GAD1	CASP8	BDNF-AS	LINC01672	SNORD15A
IL2	CCR5	ICAM1	RAC1	VCAM1	MIR7-3HG	TRP-AGG2-7
IL10	GAS5	BCL2L1	MIR34C	NF-κB1	SNORD118	TRP-AGG2-1
NOS2	CD36	IL2RA	VEGFA	PGR-AS1	LINC-ROR	SLX1A-SULT1A3
ATF6	TNF	DLEU2	CD69	NFE2L2	PIK3C2A	CHKB-CPT1B
IL6	ACE	MMP2	HIF1A	MIR451A	PRKAA1	TRP-AGG2-4
FYN	BMP2	TGFB1	TRPM7	MIR146B	SLC17A5	TRP-AGG2-8
IL5	TYR	ABCB1	CD80	MAPK1	SMAD3	TRP-AGG2-3
BAX	NOS3	SIRT1	MMP9	PPARG	SERPINE1	TRP-AGG2-6
H19	AKT1	SOD1	CXCL8	PTPN1	NAGLU	TRP-AGG2-2
IL1B						

### 3.6 The anti-inflammatory activity of APS during the PTZ-kindling process in mice

#### 3.6.1 APS treatment suppressed the activation of glia

**FIGURE 8:**
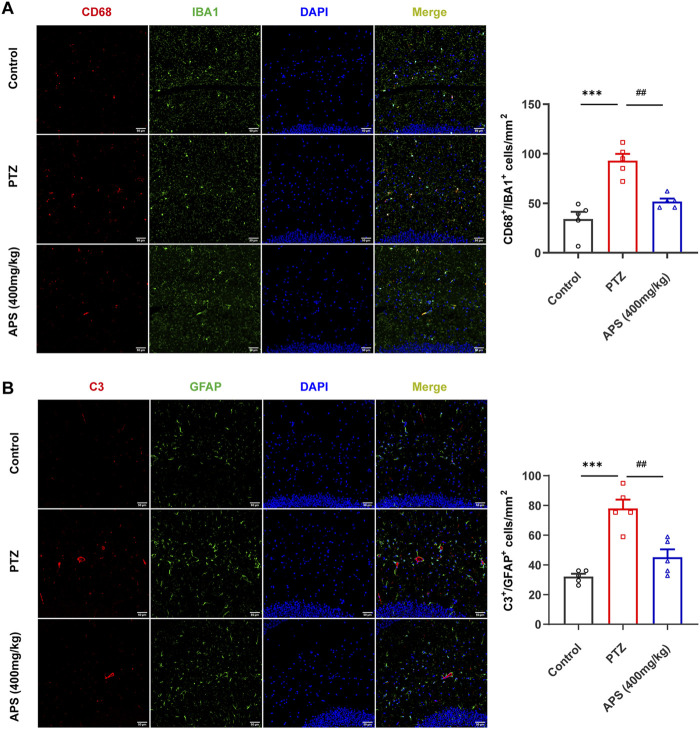
APS treatment attenuated glial activation. **(A)** Representative images depicting CD68-positive (red) microglia (IBA1, green) labeled with DAPI (blue) in the hippocampus from the control, PTZ, and APS (400 mg/kg) groups, along with quantification of CD68^+^ microglia. **(B)** Representative images showing C3-positive (red) astrocytes (GFAP, green) labeled with DAPI (blue) in the hippocampus from the control, PTZ, and APS (400 mg/kg) groups and quantification of C3^+^ astrocytes. Scale bars = 50 μm. Data were subjected to one-way ANOVA with Tukey’s *post hoc* multiple comparison test (*n* = 5 mice/group) and presented as mean ± SEM. ***: *p* < 0.001 for PTZ group *vs*. control group; ^##^: *p* < 0.01 for PTZ group *vs*. APS group.

Immunofluorescent labeling of ionized calcium-binding adapter molecule 1 (IBA1, a microglial marker) and cluster of differentiation 68 (CD68, highly expressed in activated microglia (Chen et al., 2021)) revealed an increased presence of activated microglial cells in the PTZ group mice compared to those in the control group (*p* < 0.001). In contrast, the APS (400 mg/kg) group demonstrated a notable decrease in the count of IBA1^+^/CD68^+^ cells when compared to the PTZ group (*p* < 0.01, Figure 8A). This finding implies that the administration of APS is associated with a suppressed activation of microglia in PTZ-induced kindling in mice.

Astrogliosis was characterized by an augmentation in the population of glial fibrillary acidic protein (GFAP)-positive cells, along with the co-localization of complement 3 (C3), serving as a hallmark for reactive astrocytes (Meyer et al., 2023). Immunofluorescence analyses revealed a notable escalation in the count of C3^+^/GFAP^+^ cells within the PTZ group compared to the control group (*p* < 0.001). In contrast, the APS (400 mg/kg) group exhibited a significant reduction in the number of C3^+^/GFAP^+^ cells when compared to the PTZ group (*p* < 0.01, Figure 8B), suggesting that the administration of APS is associated with a mitigated activation of astrocytes in PTZ-induced kindling in mice.

#### 3.6.2 The intervention of APS reduced the levels of pro-inflammatory cytokines

**FIGURE 9:**
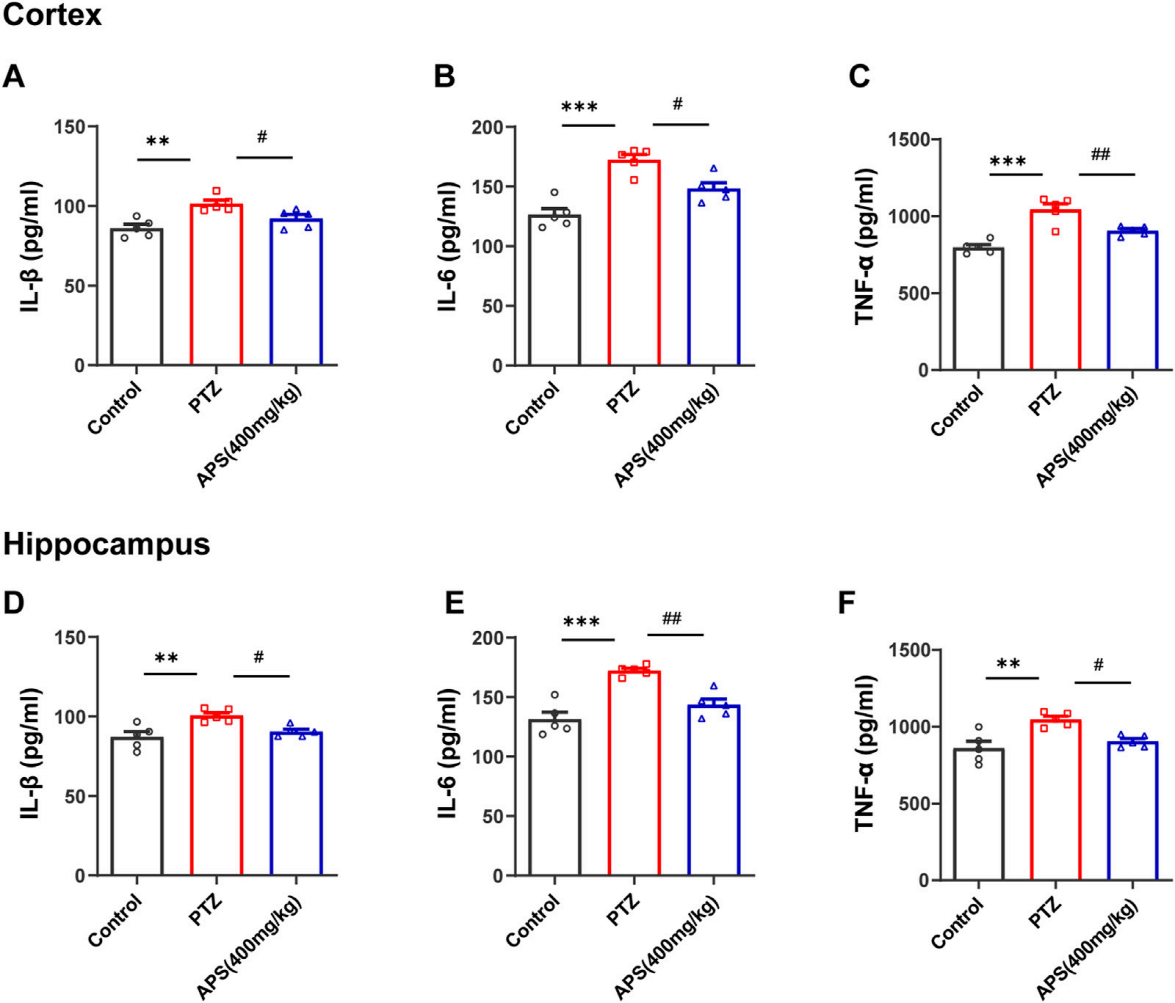
Effects of APS on the cortical and hippocampal levels of inflammatory cytokines. **(A–C)** The concentration of IL-1β, IL-6, and TNF-α in the cortex. **(D–F)** The concentration of IL-1β, IL-6, and TNF-α in the hippocampus. Data were subjected to one-way ANOVA with Tukey’s *post hoc* multiple comparison test (*n* = 5 mice/group) and presented as mean ± SEM. **: *p* < 0.01, ***: *p* < 0.001 for PTZ group *vs*. control group; ^#^: *p* < 0.05, ^##^: *p* < 0.01 for PTZ group *vs*. APS group.

Higher pro-inflammatory cytokine expression is another important feature of neuroinflammation that occurs along with glial activation (Saieva and Taglialatela, 2022). To determine the effect of APS on pro-inflammatory cytokines in PTZ-induced kindling in mice, we detected the levels of TNF-α, IL-1β, and IL-6 in the hippocampus and cortex using ELISA kits (Figure 9). The findings demonstrated a noticeable increase in these cytokines’ levels in the PTZ group when contrasted with the control group. In the APS group, the high levels of IL-1β, IL-6, and TNF-α were notably reduced following treatment, which was significantly different when compared to the PTZ group.

### 3.7 APS inhibited TLR4/NF-κB activation in the Hip

**FIGURE 10:**
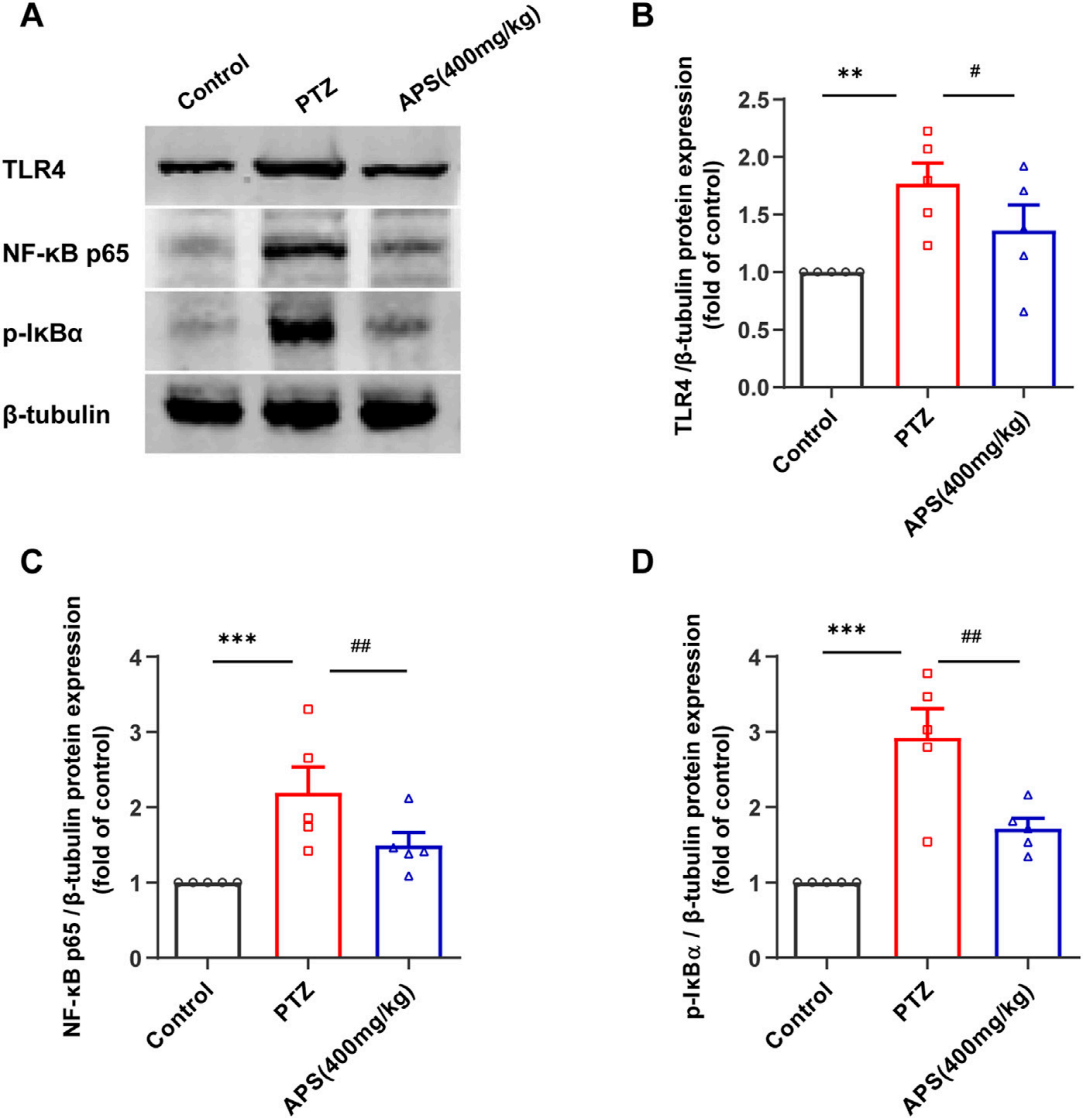
APS suppressed the activation of the TLR4/NF-κB pathway in the Hip. **(A)** Representative Western blot Images of TLR4, NF-κB p65, and p-IκBa in the Hippocampus. **(B–D)** The levels of TLR4, NF-κB p65, and p-IκBα protein expression were determined and normalized. Data were subjected to one-way ANOVA with Tukey’s *post hoc* multiple comparison test (*n* = 5 mice/group) and presented as mean ± SEM. **: *p* < 0.01, ***: *p* < 0.001 for PTZ group *vs*. control group; ^#^: *p* < 0.05, ^##^: *p* < 0.01 for PTZ group *vs*. APS group.

To affirm whether the TLR4/NF-κB signaling pathway was involved in the therapeutic mechanism of APS-treated PTZ-kindling mice, we evaluated the protein expressions of TLR4, NF-κB p65, and p-IκBα present in the Hip tissues via western blot analysis (Figure 10), corroborating the enriched results obtained from our previous network pharmacology study. Our study revealed a significant increase in the expressions of TLR4, NF-κB, and p-IκBα proteins in the Hip tissues of the PTZ group when compared to the control group ((*p* < 0.01, *p* < 0.001, Figures 10B–D). On the other hand, treatment with APS led to a marked decrease in the expressions of TLR4, NF-κB p65, and p-IκBα proteins (*p* < 0.05, < 0.01, Figures 10B–D). Our study demonstrates that APS can effectively target and inhibit the activity of the key adapter molecule TLR4, hindering signal transduction in the TLR signaling pathway, leading to the attenuation of the downstream NF-κB-mediated inflammatory response and amelioration of PTZ-kindling mice symptoms. These observations are consistent with the outcomes of our network pharmacology investigation.

## 4 Discussion

In recent years, the multifaceted and multitarget healing properties of active constituents present in Chinese herbology have captured the interest of numerous academic experts. APS is a frequently utilized component of TCM in treating neurodegenerative and neuroinflammatory diseases (Bi et al., 2020). However, there remains a lack of clinical and experimental investigations into the potential therapeutic benefits of APS on epilepsy. Our current study is the first to validate the effectiveness of APS in treating epilepsy via animal experiments that utilize mouse models. The findings indicate that administering APS prior to PTZ administration can minimize seizure severity and hinder kindling development. Furthermore, APS treatment enhanced spatial memory and recognition capabilities in mice, and brain histopathology revealed its reduced neuronal damage. The mechanism behind these effects may involve inflammation suppression via the modulation of the TLR4/NF-κB pathway.

The PTZ-induced seizure model is considered the gold standard for the rapid assessment of new anti-epileptogenic drugs using mouse models. Simultaneously, PTZ kindling remains one of the most widely used animal models for studying the neurobiology of epileptogenesis and its associated cognitive comorbidities (Singh et al., 2021). In our current investigation, we were able to validate that the repeated administration of a subconvulsive dose of PTZ triggers behavioral seizures and cognitive impairment in a PTZ-induced kindling mouse model. However, APS (400 mg/kg) significantly decreased the average seizure score compared to the PTZ group, demonstrating anti-convulsant activity. Moreover, APS treatment was found to reverse deficient memory and learning behaviors, exhibiting neuroprotective properties concerning cognition. Previous studies have provided evidence that AM extract is effective in managing acute PTZ-induced seizures in mice, while saponin AM extract has demonstrated anti-convulsant activity in rats (Aldarmaa et al., 2010; Jalsrai et al., 2010).

Meanwhile, other investigations have also found that APS can mitigate cognitive impairment in mice with Alzheimer’s disease (Huang et al., 2017; Liu Y. et al., 2019; Qin et al., 2020), implying a potentially significant role for APS in treating epilepsy and associated cognitive impairment, which aligns with the findings of our study. Interestingly, our study found that the 800 mg/kg of APS did not demonstrate the same efficacy as the 400 mg/kg dosage in the acute seizures model. This phenomenon may be attributed to the potential reduced intestinal absorption of APS at higher doses, as APS is a large molecular polysaccharide, which could lead to lower concentrations of active components in the bloodstream (Ye et al., 2023). Furthermore, high doses of APS might have adverse effects on the digestive system, leading to gastrointestinal disturbances that could impact the drug’s absorption and tolerability. In addition to behavioral changes, prolonged seizures can trigger pathological changes that cause significant neuronal loss in the hippocampal structures (Mazumder et al., 2017). Our current findings are consistent with earlier research, as we observed increased numbers of Nissl-stained dark neurons, an indicator of degraded cell bodies, in the CA1, DG, and CA3 regions of the PTZ group mice. On the other hand, APS treatment resulted in a reduced number of dark neurons, which suggests a decrease in neuronal damage likely attributable to APS’ suppression of PTZ-induced kindling. Hence, the efficacy of APS in suppressing PTZ-induced kindling supports their potential as a treatment for preventing the development of epilepsy.

TCM has demonstrated superior clinical efficacy for the treatment of epilepsy and is considered a viable alternative or supplementary treatment option primarily because of its ability to differentiate and treat various syndromes with a favorable safety profile (Yang et al., 2021). Nonetheless, due to its interactions with multiple protein targets, TCM can lead to complex pharmacological effects and pose significant challenges for drug development, thus requiring comprehensive evaluation of its safety and efficacy. Network pharmacology provides a network-based approach that predicts potential interactions and complications among biological systems, drugs, and diseases. This approach provides crucial insights into the feasibility and accessibility of TCM for treating different diseases (Nogales et al., 2022). In our study, we employed a blend of experimental verification and network pharmacology to investigate the possible anti-epileptic regulatory mechanism of APS in mice with PTZ-induced epilepsy. We constructed a PPI network and identified the top 10 core genes: IL6, TNF, IL1B, IL10, IL18, CXCL8, NF-κB1, MMP2, MMP9, and VEGFA. To gain detailed insight into the effects of APS on epilepsy and its cognitive dysfunction, we performed GO and KEGG pathway enrichment analyses. As a result, our further screening demonstrated that pro-inflammatory cytokines (PICs), such as IL-1β, IL-6, and TNF-α, and the TLR4/NF-κB pathway were associated with APS' effects in treating epilepsy.

Our investigation uncovered that the therapeutic intervention of APS exhibited a significant reduction in activation of neuroglial cells, concurrently with the suppression of key PICs, namely, IL-1β, IL-6, and TNF-α in both the Hip and cortex regions of the PTZ-induced kindling mouse model, indicating APS’ anti-inflammatory properties. Animal and human studies have highlighted the significant impact of neuroinflammation on epilepsy and its coexisting conditions (Vezzani et al., 2013; Barker-Haliski et al., 2017). Research has shown that epileptic seizures are linked to heightened levels of PICs, primarily IL-1β, IL-6, and TNF-α. These cytokines are likely to play a crucial role in the pathophysiology of seizures, resulting in neuronal hyperexcitability and rendering the brain susceptible to ictogenesis and epileptogenesis (Soltani Khaboushan et al., 2022). Our findings corroborate this statement. Furthermore, neuroinflammation is closely associated with the activity of the central nervous system’s innate immune cells, particularly microglia and astrocytes (Kwon et al., 2020). Activated microglia and astrocytes are recognized as crucial factors in the hyperexcitability of neuronal networks that underlie seizures (El-Sayed et al., 2023). This functional activation leads to alterations in the expression of intracellular metabolites, transporters, and membrane proteins (Vezzani et al., 2017). In addition, activated neuroglial cells release a diverse range of inflammatory factors that may be involved in the maintenance of tissue homeostasis or result in the development of epileptogenesis and cognitive deficits (Rong et al., 2019; Kavaye Kandeda et al., 2021). The observed activation of microglia and astrocytes in our study provided evidence that inflammatory responses mediated by glial cells fueled neuronal hyperexcitability and the development of epilepsy. The anti-inflammatory properties of APS may have potential benefits in reducing the likelihood of epileptogenesis. Our investigation indicated that APS treatment, which effectively suppresses neuroinflammation, may play a role in mitigating the severity of seizures, the development of kindling, and related cognitive impairment.

After conducting network pharmacology analyses and reviewing the relevant literature, the TLR4/NF-κB pathway was further validated in animal studies to investigate the mechanism of APS treatment in epileptogenesis and its comorbidities. The potential involvement of multiple inflammatory mediators in epileptogenesis is currently the focus of research. Toll-like receptors (TLRs), which are essential parts of the innate immune system, play a crucial role in neuroinflammation (Soltani Khaboushan et al., 2022). Many studies have suggested that the activation of TLR4 may trigger seizures (Paudel et al., 2020). Activation of TLR4 promotes activation of the NF-κB pathway, prompting the release of inflammatory mediators, such as PICs, and exacerbating the inflammatory state of the brain, which, in turn, causes increased blood-brain barrier permeability, making the brain more susceptible to seizures. Our results demonstrated a noteworthy increase in the expression levels of TLR-4 and NF-κB p65 in the PTZ-kindled mice compared to the control group. However, in mice treated with APS, the expression levels of these molecules were found to be considerably lower in hippocampal tissues. Thus, we postulated that the inhibition of the PTZ-induced TLR-4/NF-κB p65 signaling pathway activation in APS-treated mice could represent a pivotal mechanism for suppressing inflammation cytokine expression within the hippocampal tissues. Previous research studies have also indicated that APS can induce anti-inflammatory effects by modulating the TLR-4/NF-κB signaling pathway in both *in vitro* and *in vivo* settings (Lv et al., 2017; Zhou et al., 2017; Liu T. et al., 2019; Zhang et al., 2020). Accordingly, our findings reveal that the modulation of the TLR4/NF-κB pathway by APS administration leads to a reversal of PTZ-induced seizures and a reduction in neuroinflammation.

This study has several limitations. Some target genes related to drugs and diseases may have yet to be included in the publicly available databases, which can be a limitation of network pharmacology studies. Moreover, to confirm the accuracy and reliability of the results, further *in vivo* and *in vitro* experimental validation is required.

## 5 Conclusion

Our study outcomes demonstrate that APS confer superior protective effects against PTZ-induced kindling, cognitive dysfunction, and histopathological changes. The neuroprotective properties of APS may be attributed to their modulation of the TLR4/NF-κB signaling pathway, resulting in a decrease in neuroinflammation. These results underscore the possibility of a novel therapeutic strategy for treating epileptogenesis and associated cognitive deficits.

## Data Availability

The original contributions presented in the study are included in the article/Supplementary Material, further inquiries can be directed to the corresponding author.
