# Supplementary material for: Astragalus polysaccharides ameliorate epileptogenesis, cognitive impairment, and neuroinflammation in a pentylenetetrazole-induced kindling mouse model
Source: Front Pharmacol. 2024 Feb 9;15:1336122. doi: 10.3389/fphar.2024.1336122 (PMC10884767; doi:10.3389/fphar.2024.1336122)
Supplement: Supplementary file 1 [file DataSheet1.ZIP › Supplementary Figure1/Supplementary_Material.docx]

Supplementary Material

Astragalus Polysaccharides Ameliorate Epileptogenesis, Cognitive Impairment, and Neuroinflammation in a Pentylenetetrazole-Induced Kindling Mouse Model

Yuling Lu ^1†^, Minglin Lin ^2†^, Sijie Ou ^1^, Lanfeng Sun ^1^, Kai Qian ^1^, Huimin Kuang ^1^, Yuan Wu ^*^

*** Correspondence:** Yuan Wu, nwuyuan90@163.com

# Supplementary Figure1


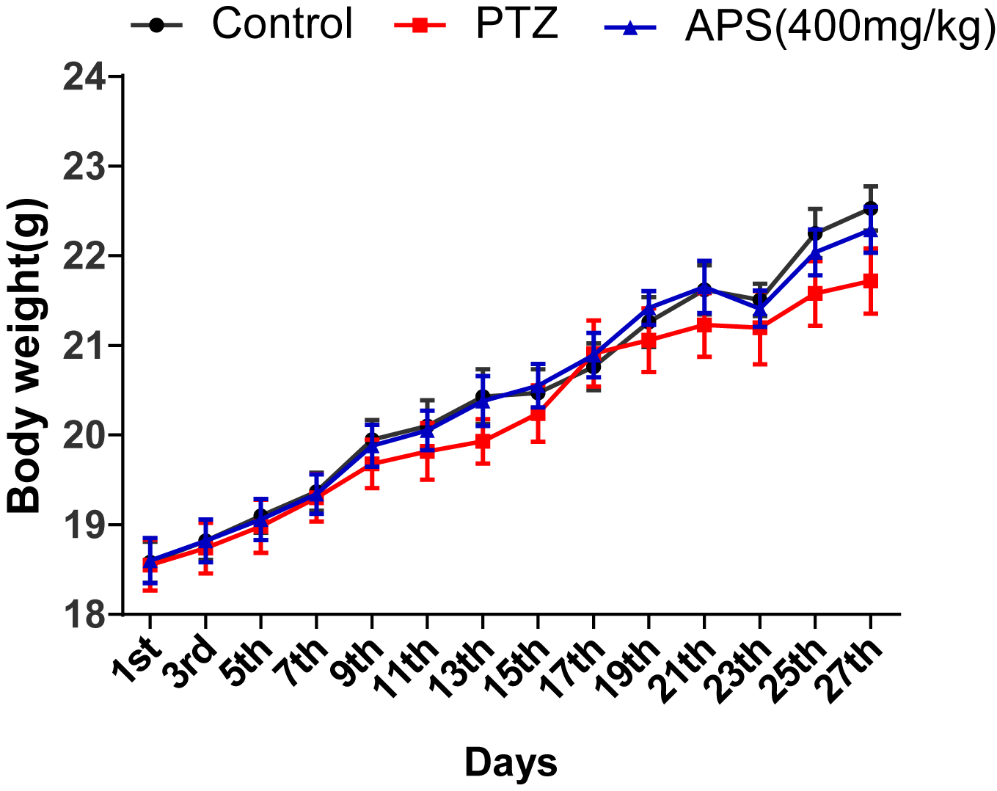


**Supplementary Figure 1.** Body weight of mice during the PTZ-kindling process. The administration of 400 mg/kg APS had no significant impact on the body weight of mice compared with the control and PTZ group.
