# Supplementary figures and images for: Astragalus polysaccharides ameliorate epileptogenesis, cognitive impairment, and neuroinflammation in a pentylenetetrazole-induced kindling mouse model
Source: Front Pharmacol. 2024 Feb 9;15:1336122. doi: 10.3389/fphar.2024.1336122 (PMC10884767; doi:10.3389/fphar.2024.1336122)

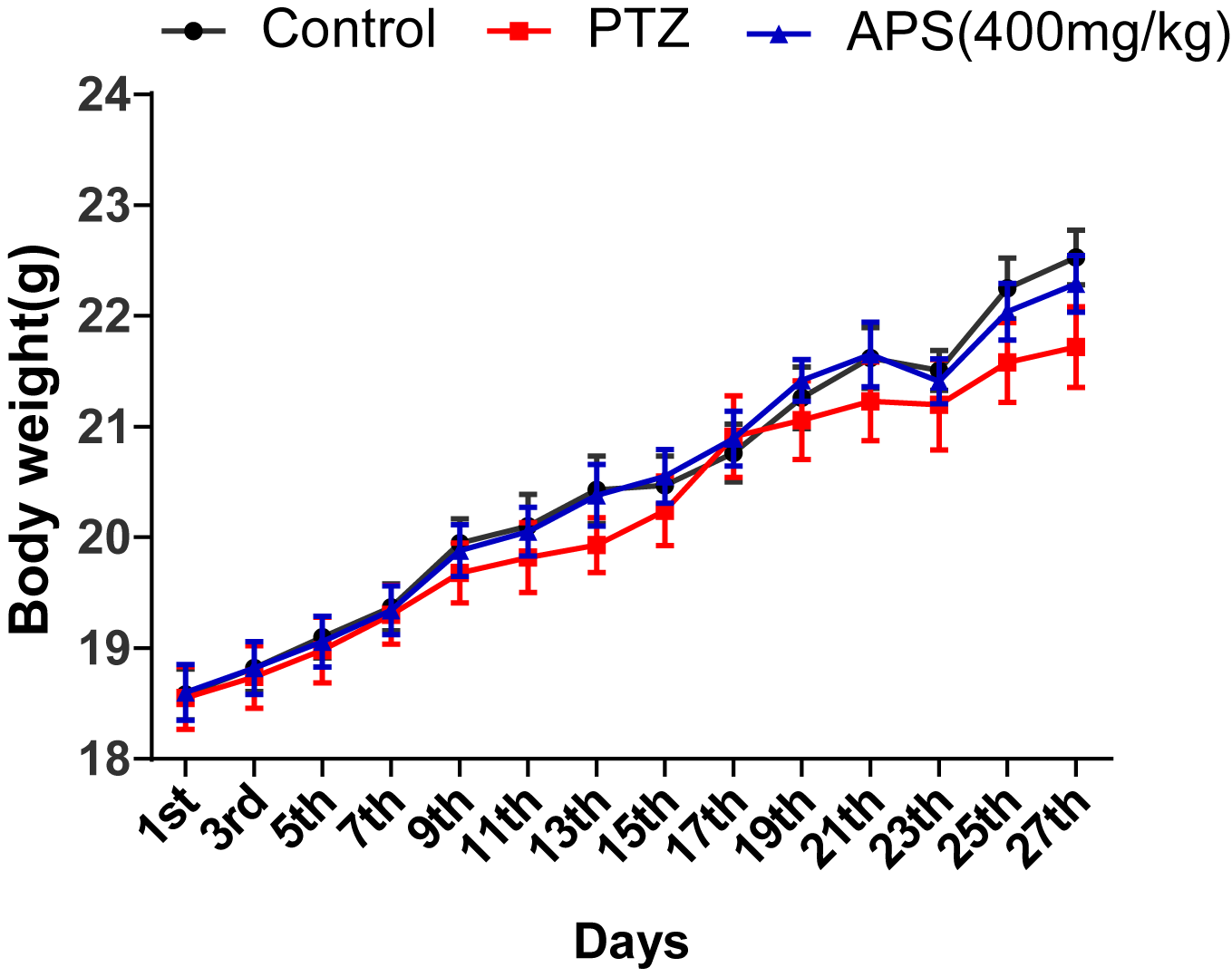

Supplement: Supplementary file 1 [file DataSheet1.ZIP › Supplementary Figure1/Supplementary Figure1.tif]
